# Examination of Genetic Control Elements in the Phototrophic Firmicute *Heliomicrobium modesticaldum*

**DOI:** 10.3390/microorganisms10050876

**Published:** 2022-04-22

**Authors:** Alexandria M. Layton, Kevin E. Redding

**Affiliations:** Center for Bioenergy and Photosynthesis, School of Molecular Sciences, Arizona State University, Tempe, AZ 85287, USA; amlayto1@asu.edu

**Keywords:** Heliobacteria, promoters, reporters, gene expression, phototrophic bacteria

## Abstract

*Heliomicrobium modesticaldum* has been used as a model organism for the Heliobacteria, the only phototrophic family in the Firmicutes. It is a moderately thermophilic anoxygenic phototrophic bacterium that is capable of fermentative growth in the dark. The genetic manipulation of *H. modesticaldum* is still in its infancy. Methods to introduce genes through the use of exogenous plasmids and to delete genes from the chromosome through the use of the native CRISPR/Cas system have been developed in the last several years. To expand our genetic toolkit, it was necessary to control gene expression. In this study, we analyzed constitutive and inducible promoters developed for clostridia for their use in *H. modesticaldum* and further tested two reporters, *adhB* and *lacZ*, as indicators of promoter strength. Alcohol dehydrogenase (AdhB) was unsuitable as a reporter in this species due to high endogenous activity and/or low activity of the reporter, but a thermostable LacZ worked well as a reporter. A set of constitutive promoters previously reported to work in *Clostridium thermocellum* was found to be reliable for controlling the expression of the *lacZ* reporter gene in *H. modesticaldum* at a range of activities spanning an order of magnitude. An anhydrotetracycline-inducible promoter was created by inserting *tetO* operators into a strong constitutive promoter, but it was not fully repressible. The implementation of a xylose-inducible promoter resulted in complete repression of β-gal in the absence of xylose, and reliable expression tunable through the concentration of xylose added to the culture.

## 1. Introduction

The Heliobacteria are the only phototrophic members of the phylum Firmicutes. Although unable to fix inorganic carbon, they are capable of growing phototrophically (using light as an energy source) via electron transport driven by their photochemical reaction center (RC) and chemiosmotic ATP production, or chemotrophically through fermentation of a suitable carbon source. In the case of *Heliomicrobium* (previously *Heliobacterium*) *modesticaldum*, this is primarily pyruvate or lactate, but other species can use a greater range of organic molecules [1,2,3]. This metabolic flexibility renders phototrophy optional and allows for genetic manipulation of components of the phototrophic apparatus without ill consequences. This, combined with the simplicity of their phototrophic apparatus, make Heliobacteria ideal candidates not only for studying the fundamental mechanisms and evolution of phototrophy but also for studying other aspects of metabolism [4,5,6].

*H. modesticaldum* is a moderate thermophile of the family Heliobacteriaceae and has been used as a model system for this group of organisms. Since its discovery and isolation from Icelandic volcanic soil in 1995 [2], its genome has been fully sequenced [7], transcriptomes have been reported [8], and methods have been developed for the introduction of plasmids via conjugation from *Escherichia coli* [9]. A method to leverage its endogenous CRISPR/Cas system to perform precise chromosome editing [9,10] now allows for reverse genetics to be applied to this organism [11,12,13,14]. However, one tool that is lacking is the ability to control and fine tune gene expression. The purpose of this report is to remedy this deficiency.

*H. modesticaldum* has displayed stringency when it comes to genetic elements and manipulation. To introduce non-native plasmids, the plasmids must first be pre-methylated at presumed restriction endonuclease sites and then conjugated using an engineered *E. coli* host; without the methylation, the DNA will be cleaved and destroyed upon insertion into the cells [9]. In addition, while the Cas9 system from *Streptococcus pyogenes* could be adapted for use in other organisms, *H. modesticaldum* could not tolerate it. Similar to other clostridia [15], the organism’s endogenous CRISPR/Cas system was employed to perform gene deletions and chromosome editing [10]. Both of these genetic manipulation techniques were developed by adapting techniques used for other members of the Clostridiales. We took the same route to find methods of controlling gene expression in *H. modesticaldum*.

In this study, we analyzed a series of promoters used in Clostridia for their activity in *H. modesticaldum*. A set of constitutive *C. thermocellum* promoters previously analyzed by Olson et al., 2015 [16] was chosen for several reasons: *C. thermocellum* is a member of the Clostridiales family much like *H. modesticaldum*, *C. thermocellum* grows at a similar temperature to *H. modesticaldum*, the promoters are orthogonal and therefore should not affect the activities of native genes, and these promoters were already previously characterized in *C. thermocellum* and *E. coli*. We then utilized several of the constitutive promoters to attempt to construct an anhydrotetracycline inducible promoter. We have also tested a xylose-inducible promoter. In addition, as a result of this study, two reporters, alcohol dehydrogenase and β-galactosidase, were tested in *H. modesticaldum* for the first time.

## 2. Materials and Methods

### 2.1. Strains and Culture Conditions

*E. coli* strains (TOP10 for activity assays, s17-1 for conjugations) were grown in Luria-Bertani (LB) broth (BD Difco, Franklin Lakes, NJ, USA). When needed, the antibiotics erythromycin (Sigma-Aldrich, St. Louis, MO, USA) and chloramphenicol (Acros, Geel, Belgium) were added to a final concentration of 100 µg mL^−1^ and 15 µg mL^−1^, respectively. Cultures were grown overnight at 37 °C while shaking at 250 rpm.

All work utilizing *H. modesticaldum* was performed in an anaerobic chamber (Coy Laboratory Products, Grass Lake, MI, USA), with an atmosphere of 20% CO_2_ and 2–4% H_2_, and balance N_2_. *H. modesticaldum* strains were grown in pyruvate yeast extract (PYE) medium following the recipe and procedure outlined previously [9]. When necessary, erythromycin was added to a final concentration of 10 µg mL^−1^. Cultures were grown at 50 °C under 790-nm light-emitting diodes (LEDs) (Marubeni America, New York, NY, USA) at a flux of 30 µmol photons m^−2^ s^−1^.

### 2.2. Plasmid Construction

All constructs were made using the vector pMTL86251 [17]. Plasmids containing promoter regions originating from *C. thermocellum* and thermostable *lacZ* and *adhB* reporter genes were gifts from Daniel Olson (Dartmouth College) [16]. Promoter sequences are listed in Table S1. All oligonucleotide primers were purchased from Integrated DNA Technologies (Coralville, IA, USA) and are listed in Table S2. Restriction enzymes and Q5 DNA polymerase used during plasmid construction were purchased from New England Biolabs (Ipswich, MA, USA). 

The seven promoters (gapDH, eno, cbp_2, 2926, 0815, 0966, and 2638) and two reporters (*adhB*, *lacZ*) were PCR amplified individually from the plasmids provided by Daniel Olson (pDGO89, pDGO95, pDGO102, pDGO105, pDGO106, pDGO108, and pDGO117, listed in Table S3) using primers containing restriction sites (PspOMI upstream and BamHI downstream for promoters, BglII upstream and SalI downstream for reporters). The PCR products were digested with their respective enzymes then ligated into pMTL86251 at the NotI and XhoI sites using a modified Golden Gate assembly protocol. Modifications to the protocol include that the ligation mixture contained NotI and XhoI restriction enzymes in place of a Type IIS enzyme, and heat cycles were set up as follows: 10 min at 37 °C then 10 min at 16 °C, repeated 10 times, followed by 85 °C for 20 min, then held at 4 °C. The module containing the gene coding for *xylR* in the reverse orientation from the promoter region containing the *xylO* sequence (P_xyl_) was synthesized by GenScript (Piscataway, NJ, USA) with a NotI site downstream of the *xylR* and a BamHI site downstream of P_xyl_ and cloned into pMTL86251 at those sites. Once received from Genscript, the *lacZ* reporter (amplified using the same primers as before, adding BglII and SalI sites) was cloned into this plasmid at the BamHI and XhoI sites, resulting in pALX3. Constructs were confirmed by Sanger sequencing.

To add the *tetO1* sequences, the P_gapDH_—*lacZ* module was amplified from plasmid pAL66 and inserted into pUC19 using BamHI and SalI sites. The *tetO1* sequences were inserted using primers that amplified around pUC19 to generate a single linear product and ligated (recircularized) using Genscript’s GenBuilder Cloning Kit. After confirming the *tetO* sequences through the use of Sanger sequencing, the P_gapDH/*tetO*_—*lacZ* module was amplified from the pUC19 using primers to add BglII (upstream of P_gapDH/*tetO*_) and SalI (downstream of *lacZ*) sites. The *tetR* gene was amplified from genomic preps of XL1-Blue *E. coli* cells using primers to add PspOMI (downstream) and EcoRI (upstream) sites, and the eno promoter was amplified using primers to add EcoRI (downstream) and BglII (upstream) sites. All three products (P_gapDH/*tetO*_—*lacZ*, *tetR*, and eno) were digested with their respective enzymes and inserted into pMTL86251 at the NotI and XhoI sites using the modified Golden Gate assembly process described earlier.

Plasmids were transformed into *E. coli* TOP10 and s17-1 using standard techniques and were further conjugated into *H. modesticaldum* using the procedure described previously [9].

### 2.3. Induction Tests

Anhydrotetracycline (aTc) (Sigma-Aldrich) was prepared in ethanol (10 µg mL^−1^) and added to the appropriate final concentration (ranging from 0 to 200 µg L^−1^) when the cells entered the log phase (OD_735_ ≈ 0.5). Cells were assayed after induction for 3 h. For some growth tests, aTc was added at the time of inoculation. For all xylose induction tests, cells were grown in PYE media, with xylose (Sigma-Aldrich) added at the time of inoculation.

### 2.4. Cell Free Extracts

Cell free extracts were obtained following the protocol outlined previously [4], with a few alterations. *E. coli* and *H. modesticaldum* cells were harvested by centrifugation at 5000× *g* for 15 min. Cells were washed and resuspended in 20 mM Tris-HCl buffer pH 8.0 (Santa Cruz Biochemicals, Dallas, TX, USA) containing 100 µL of 3 mg mL^−1^ lysozyme (Goldbio, St. Louis, MO, USA), then incubated at 37 °C for 20 min. Phenyl-methanesulfonyl fluoride (PMSF) (Sigma-Aldrich) was added to 1 mM, and the suspension was sonicated in an Aquasonic sonication bath (Model 75T, VWR, Scientific Products, Radnor, PA, USA) for 20 min with minute-long pauses every 5 min. The lysate was centrifuged at 20,000× *g* for 30 min, and the pellet was discarded. Protein concentration in the extracts was determined using the Pierce BCA protein assay kit (Thermo Scientific, Waltham, MA, USA). 

### 2.5. Reporter Activity Assays

Activity assays were modeled after those described previously [16]. All assays were performed using a 96-well plate and read using an Epoch Microplate spectrophotometer (BioTek Instruments, Inc., Winooski, VT, USA). All reagents used for the assays were purchased from Sigma-Aldrich (St. Louis, MO, USA).

#### 2.5.1. Alcohol Dehydrogenase Assays

In this assay, 4 mM acetaldehyde and 0.4 mM NADPH were added to extract (50 µg of protein) in 100 mM Tris-HCl buffer (pH 7.6), and the absorbance at 340 nm was read every 7 s over a period of 10 min to detect the rapid loss of NADPH. 

#### 2.5.2. β-Galactosidase Assays 

To measure β-galactosidase (β-gal) activity, 50 mM β-mercaptoethanol, 1 mM MgCl_2_, and 0.66 mg mL^−1^ ONPG were added to 50 µg of protein in 100 mM sodium phosphate buffer (pH 7.5). The absorbance at 420 nm was immediately read, and then the plate was incubated at 37 °C. Absorbance was read after 30, 60, and 90 min of incubation to calculate a linear rate of activity corresponding to the cleavage of ONPG. The increase in A_420_ was converted into units (U) by using the extinction coefficient of ONP (ε = 4500 M^−1^ s^−1^) and further to calculate the nmols of ONP produced per second of the reaction. 

### 2.6. Calculations—Curve Fittings and Error Calculations

Growth data were fit to the logistic equation as described [13]. β-gal activity data (product vs. time) were fit to a linear equation. The pro Fit software package (QuantumSoft; http://www.quansoft.com/, accessed on 20 March 2022) was used to produce all fittings. 

## 3. Results

To develop a gene expression system, two enzymes were tested for use as reporters in *H. modesticaldum*. Alcohol dehydrogenase and β-galactosidase have been widely used as reporters and have been tested in many bacterial species, including thermophilic Clostridia [16]. The constitutive and inducible promoters chosen for use in *H. modesticaldum* have been tested in other clostridia, including *C. thermocellum* [16], *Clostridium perfringens* [18], and *Clostridium acetobutilicum* [19].

### 3.1. Testing of Reporters in H. modesticaldum

**Figure 1:**
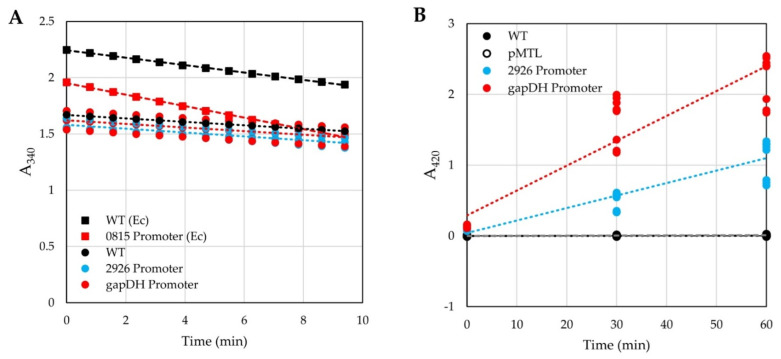
A reporter expression study utilizing two different reporters in *H. modesticaldum* and *E. coli*. Alcohol dehydrogenase (**A**) and β-gal (**B**) activities in extracts from unmodified (WT) and *H. modesticaldum* transformed with empty vector (pMTL) or expression plasmids using the 2926 or gapDH promoters. As a positive control, assays using extracts from *E. coli* (Ec) cells that were either unmodified (WT) or transformed with a vector using the 0815 promoter are included for comparison in panel (**A**). Each point represents a single technical or biological replicate. *E. coli* and WT *H. modesticaldum* controls in (**A**) are displayed with a single replicate (*n* = 1), while 2926 and gapDH samples display each individual replicate (*n* = 9); all samples in (**B**) are displayed with each individual replicate (*n* = 9). Dotted or dashed lines represent the trendline generated for each dataset.

The reporter gene *adhB* (encoding an alcohol dehydrogenase from *Thermoanaerobacter pseudethanolicus*) was cloned behind several clostridial promoters that had been previously tested in *C. thermocellum* [16]. The expression of AdhB was tested in both *H. modesticaldum* and *E. coli* by measuring alcohol dehydrogenase activity in cellular extracts after the addition of acetaldehyde and NADPH. The reduction of acetaldehyde to ethanol was monitored by the decrease of absorption of NADPH at 340 nm (Figure 1A). Wild-type (WT) *E. coli* cells displayed a higher level of basal alcohol dehydrogenase activity than WT *H. modesticaldum* cells (−0.55 mAU_340_ s^−1^ vs. −0.25 mAU_340_ s^−1^, respectively). *E. coli* transformants harboring the plasmid containing the *adhB* reporter gene under the control of promoter 0815 had significantly more alcohol dehydrogenase activity (−0.85 mAU_340_ s^−1^), an almost two-fold increase from WT expression levels. In contrast, cellular extracts of *H. modesticaldum* transformants containing plasmids using several different promoters to drive *adhB* expression exhibited activities that were barely distinguishable from WT (−0.26 or −0.28 mAU_340_ s^−1^ for gapDH and 2926 promoters, respectively (Figure 1A)). 

The *lacZ* gene from *Geobacillus stearothermophilus* [20], which encodes a thermostable beta-galactosidase (β-gal), was cloned behind several clostridial promoters. The resulting plasmids were introduced into *H. modesticaldum,* and cell-free extracts were prepared from them. β-gal activity was assayed by the addition of extract to an assay mixture containing *o*-nitrophenylgalactoside (ONPG); cleavage of ONPG into *o*-nitrophenol (ONP) and galactose was monitored by an increase in the absorbance of ONP at 420 nm. Cellular extracts from WT *H. modesticaldum* cells displayed no detectable β-gal activity (~0.05 µAU_420_ s^−1^; R^2^ = 0.1). In contrast, extracts from two *H. modesticaldum* transconjugants with promoters driving *lacZ* expression displayed much higher levels of β-gal activity: 0.6 mAU_420_ s^−1^ (gapDH promoter) and 0.3 mAU_420_ s^−1^ (2926 promoter) (Figure 1B). In all subsequent studies, the *G. stearothermophilus lacZ* gene was used as the reporter, and β-gal activities were measured over a time of 90 min to generate a linear regression curve and estimate the rate of ONP generation. Throughout this report, β-gal activity is described as units (U, nmol of ONP formed per second of reaction) per mg of protein.

### 3.2. Testing of Constitutive Promoters from C. thermocellum in H. modesticaldum

**Figure 2:**
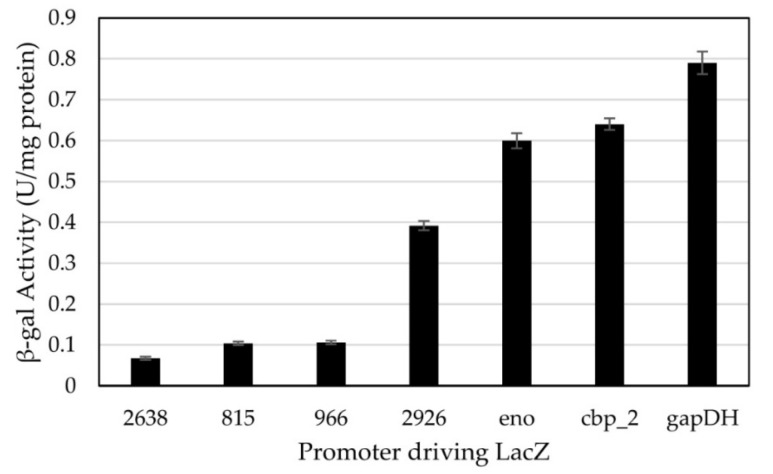
β-gal activities resulting from the expression of *lacZ* driven by different promoters in *H. modesticaldum* transformants. Units represent the nmoles of ONP formed per second of reaction. Data are represented as the average rate of three biological samples, with error bars representing the standard error.

Seven promoters from *C. thermocellum* [16] were assayed for their ability to drive expression of *lacZ* in *H. modesticaldum*. Measured activities ranged from 0.067 to 0.79 U/mg protein, with the tested promoters forming two distinct groups (Figure 2). Use of promoters 2926, eno, cbp_2, and gapDH resulted in higher expression of β-gal in *H. modesticaldum*, with cellular activities ranging from 0.39 to 0.79 U/mg protein. Use of promoters 2638, 0815, and 0966 resulted in lower expression (0.067 to 0.11 U/mg protein). 

**Figure 3:**
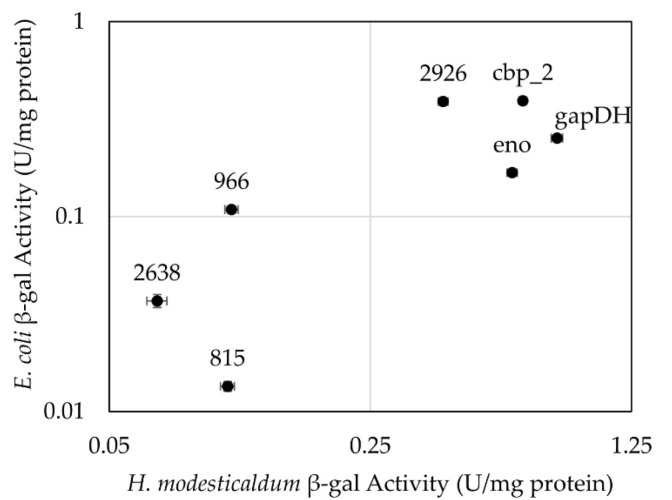
β-gal activities in *E. coli* and *H. modesticaldum* transformants plotted on a logarithmic scale. Data points are representative of the average rate of β-gal activity for each promoter. Error bars represent the standard error. (Values listed in Table S4).

Because introducing plasmids into *H. modesticaldum* requires a conjugation step from *E. coli*, it is helpful to know the activities of the promoters in both bacterial species. Therefore, activities of *E. coli* Top10 transformants harboring the same plasmids were also measured. Overall, activities resulting from the promoters in the two species displayed a positive correlation (R^2^ = 0.486), indicating that if expression of *lacZ* driven by a given promoter is low or high in one species, it will tend to be similarly low or high in the other (Figure 3). 

### 3.3. Conversion of a Constitutive Promoter into an Anhydrotetracycline-Inducible Promoter

At the start of these studies, there was no prior knowledge of which inducible systems would work in *H. modesticaldum*. We decided to construct such a system based off a system previously reported for *C. acetobutylicum* [19], involving the insertion of an orthogonal operator into one of the clostridial promoters previously tested. This system is based on the TetR/*tetO* tetracycline resistance operon. In this system, the TetR repressor binds to the *tetO* operator, inhibiting the transcription of the downstream gene (originally coding for a protein conferring resistance) [21,22]. The binding of tetracycline (Tc) to the TetR homodimer results in a conformational change, removing it from the operator and alleviating repression. As we know that *H. modesticaldum* is sensitive to Tc [9], we expect that it (and related derivatives) are able to enter the cell. Anhydrotetracycline (aTc) has a lower affinity for the ribosome than Tc, making it a weaker antibiotic, but has a higher affinity for the TetR protein, making it a better inducer [23].

**Figure 4:**
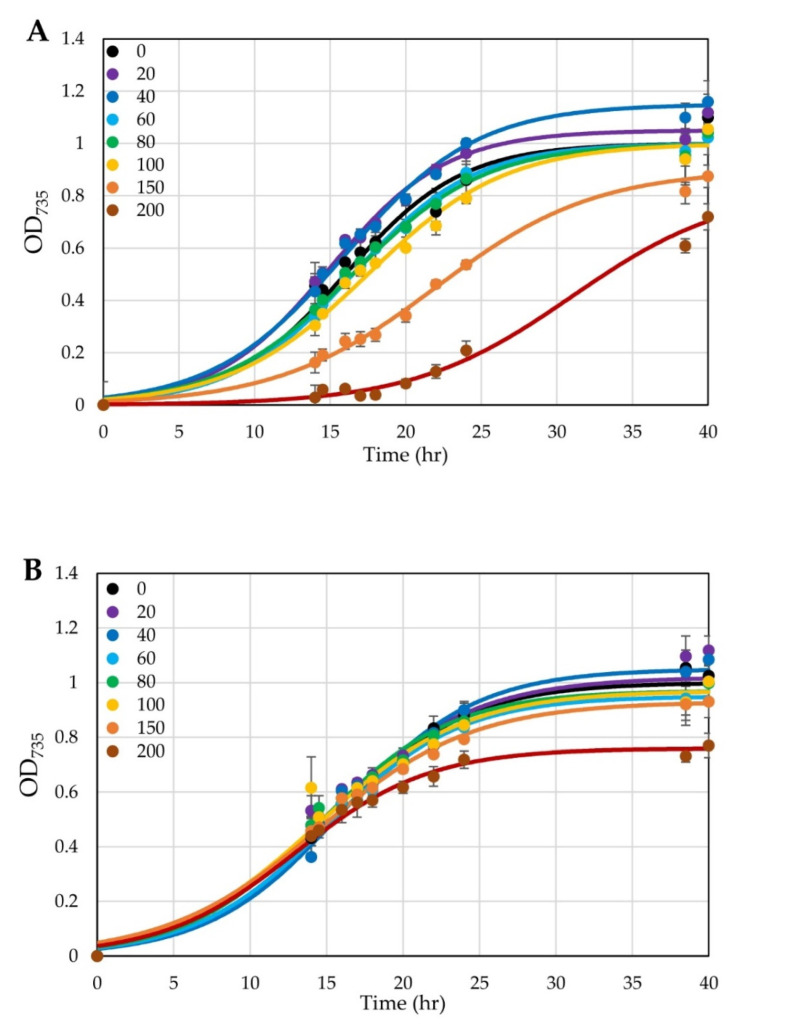
The effect of aTc on the growth of WT *H. modesticaldum*. Tested aTc concentrations ranged from 0 to 200 µg L^−1^, as indicated in the legends; aTc was added either at the time of inoculation (**A**) or at 14.5 h (**B**). Each point represents the average of three replicates; error bars represent a standard deviation. Curves represent fittings to a logistic function [13].

We characterized the effects of aTc on the growth of untransformed *H. modesticaldum* cells in liquid cultures and found that *H. modesticaldum* can tolerate up to 100 µg L^−1^ aTc without noticeable effects on growth. In addition, *H. modesticaldum* cells can tolerate higher levels of aTc (up to 150 ng mL^−1^) if the aTc is added after the cells have grown to an O.D. of about 0.5 (Figure 4). These levels are slightly lower than those tolerated by *C. acetobutylicum* [19]; however, they are also only marginally higher than the levels at which *H. modesticaldum* can tolerate Tc, which has a minimal inhibitory concentration (MIC) of 200 µg L^−1^ on agar plates [1]. 

**Figure 5:**
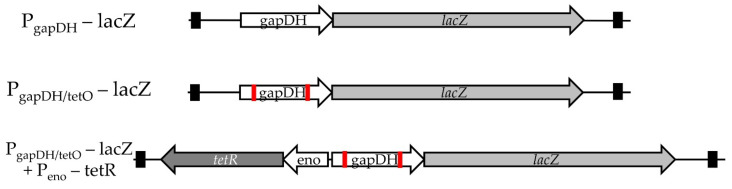
The schematics of each construct made to test the aTc inducible system. P_gapDH_—*lacZ* is the starting point: the P_gapDH_ promoter driving the expression of *lacZ* (plasmid pAL66). P_gapDH/*tetO*_—*lacZ* represents the insertion of a *tetO1* box into each −35/−10 region of the gapDH promoter (pAL88). P_gapDH/*tetO*_—*lacZ* + P_eno_—*tetR* represents the addition of the *tetR* gene driven by P_eno_ in the opposite orientation to the P_gapDH/*tetO*_—*lacZ* gene (pAL111). White arrow boxes represent promoter sequences, while gray arrow boxes are open reading frames. Solid red boxes represent the *tetO1* boxes. Solid black boxes represent the terminators present at either end of the MCS in plasmid pMTL86251.

**Figure 6:**
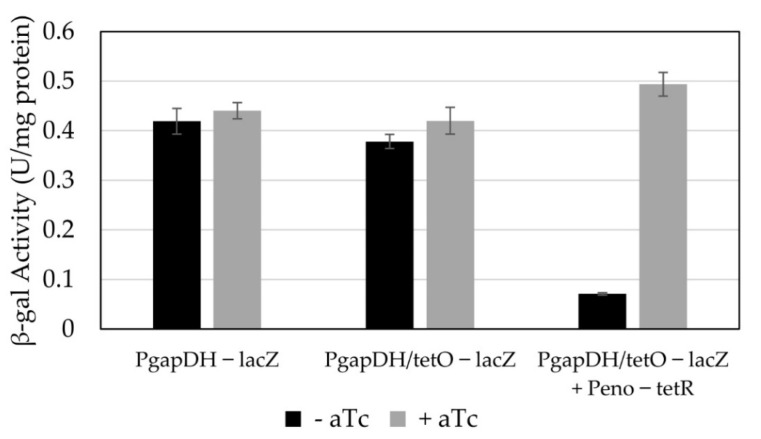
Anhydrotetracycline-inducible promoter construct plasmids and their associated β-gal activity in cultures with and without the addition of aTc (150 µg L^−1^). The values represent the average specific activity in technical triplicates for P_gapDH_—*lacZ* and P_gapDH/*tetO*_—*lacZ* controls (*n* = 3), and the average rate of ONP production of biological and technical triplicates of the P_gapDH/*tetO*_—*lacZ* + P_eno_—*tetR* constructs (*n* = 9). The error bars represent the standard deviation of the sets.

We chose the constitutive promoter associated with the highest β-gal expression in *H. modesticaldum*—gapDH—as the starting point for an aTc-inducible system. The gapDH promoter has two annotated −35/−10 regions [16]; therefore, the *tetO* sequence *tetO1* [22] was added to both sites (Figure 5). The replacement of the 15- or 17-bp spacer region between the −35 and −10 boxes with the 18-bp *tet* operator resulted in only a ~7% drop in β-gal expression (Figure 6). 

The *tetR* gene was amplified from *E. coli* and placed behind the next strongest clostridial promoter (eno) directly upstream and in the reverse orientation of the P_gapDH/*tetO*_*—lacZ* module. The expression of TetR driven by the eno promoter resulted in a ~6-fold reduction of β-gal activity, indicating that it was functioning as a repressor (Figure 6). We then tested the ability of aTc to alleviate the repression due to TetR. A concentration of 150 µg L^−1^ aTc was sufficient to fully alleviate TetR-dependent repression (Figure 6). 

### 3.4. Testing Use of a Xylose-Inducible Promoter System in H. modesticaldum

**Figure 7:**
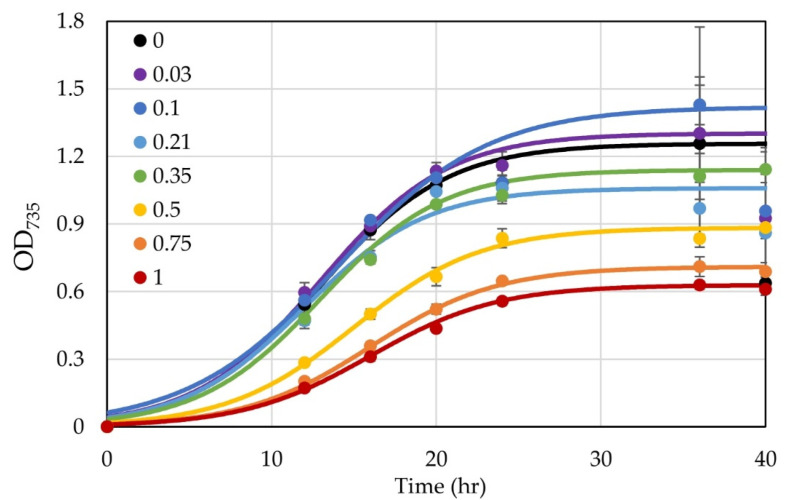
The growth of *H. modesticaldum* with varying concentrations of xylose from 0 to 1 %. Xylose concentrations (%) are displayed in the legend. Data points represent the average, and error bars represent the standard deviation of the biological triplicate (*n* = 3). Curves represent fittings of points to the logistic function [13].

We also tested the efficacy of a previously developed xylose-inducible promoter [18] in *H. modesticaldum.* It was unclear if this could work as this species is unable to use any carbohydrates as a carbon or energy source, so we did not know if the cells could take up xylose [2,4]. Similar to the aTc system, the xylose-induced system involves an operator (*xylO*) within the promoter region, which will be bound by the repressor XylR in the absence of xylose. Once xylose is introduced, it will bind to the XylR, which will dissociate from the operator, allowing for the transcription of the downstream gene of interest [18]. We first tested the effect of xylose upon the growth of untransformed *H. modesticaldum*. At xylose concentrations below 0.35%, there was no discernible inhibition of growth, but at concentrations of 0.5% and higher, growth was noticeably inhibited (Figure 7). The origin of this effect is unclear at this time, but similar effects have been seen when growing *H. modesticaldum* with other carbohydrates. If pyruvate was removed from the medium, the cells were unable to grow on 0.35% xylose, indicating that xylose does not serve as a carbon source, as expected.

**Figure 8:**
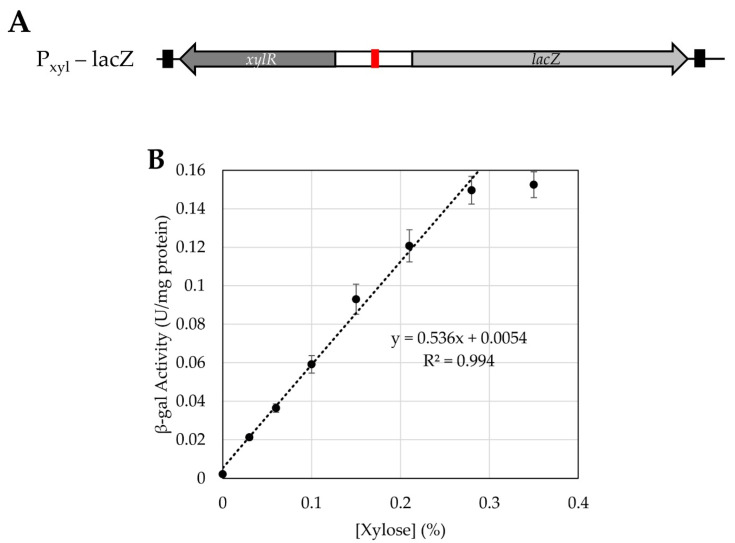
Xylose inducible promoter gene set-up (**A**) and promoter activity tuned by concentration of xylose present in media (**B**). In the schematic (**A**), the red box represents the operator, *xylO*, present in the promoter for the *lacZ* reporter. The *xylR* gene encoding the repressor is upstream and in the opposite orientation of this site; it is expressed constitutively. Terminators are present at either end of this sequence, represented as black boxes. In (**B**), specific activity is reported as β-gal units per mg protein versus xylose concentration (*w*/*v*). Each point represents the average of a biological and technical triplicate (*n* = 9), and error is represented as standard error.

The P_xyl_ promoter with the *xylR* gene (based on genes from the operon from *Clostridioides difficile*) was cloned in front of *lacZ* to make plasmid pALX3. *H. modesticaldum* transconjugants harboring pALX3 were grown with a range of xylose concentrations, and cellular extracts were prepared from log-phase cells. We found a strikingly linear correlation (R^2^ = 0.944) between xylose concentration and β-gal activity between 0.03 and 0.3% xylose (Figure 8). The activities ranged from 0.02 to 0.16 U/mg protein. These activities place this promoter at the lower end of the strength scale. Concentrations of xylose above 0.3% yielded similar or lower promoter activity, with a peak around 0.5% xylose (Figure S1). This is most likely due to the negative effects of xylose upon growth.

## 4. Discussion

### 4.1. Reporters

From the alcohol dehydrogenase activities assays, we see that *H. modesticaldum* displays some endogenous alcohol dehydrogenase activity, which could have masked the exogenous alcohol dehydrogenase activity as a result of the introduced promoters. However, the alcohol dehydrogenase assays performed with *E. coli* did show a marked difference in activity as a result of the exogenous AdhB, despite also having an active endogenous alcohol dehydrogenase (AdhE). Therefore, the lack of detectable inducible alcohol dehydrogenase activity in *H. modesticaldum* suggests that the exogenous AdhB is inactive or very weakly active in this context. For either of these reasons—the presence of endogenous activity or the lack of detectable exogenous activity—*adhB* is useless as a reporter gene for *H. modesticaldum*.

In contrast, the thermostable β-galactosidase reporter was easily measured and could be used to indicate differences in promoter strength in *H. modesticaldum*. There is negligible endogenous β-gal activity displayed by WT or empty-vector-transformed *H. modesticaldum* samples, indicating that all measured β-gal activity is a result of the promoter driving gene expression. However, we did find evidence that β-galactosidase expression had a negative effect on growth when expressed at high levels (e.g., the under control of the gapDH promoter, which had the highest activity). While β-gal is not a perfect reporter, since it does trigger these negative growth effects, it is the one reporter we were able to find during the duration of this study that provided clear, measurable differences in *H. modesticaldum* conjugants. Unfortunately, we are unable to use other more common fluorescent reporters, such as GFP (which also requires oxygen for fluorophore synthesis), due to quenching by the endogenous pigments. We will continue to analyze other reporters for their use in *H. modesticaldum*, focusing on those that could be used for real-time in vivo monitoring.

### 4.2. Constitutive Promoters

Using β-galactosidase as a reporter, we were able to determine the relative activity of a set of clostridial promoters in *H. modesticaldum*. The promoters covered a range of strengths, making up two distinct groups—high and low strength—while also displaying many differences amongst key sequence elements. If one focuses on the ribosome binding sites, several strong promoters (cbp_2, gapDH, and 2926) share a similar sequence (AGGAGG). However, a weak promoter (2638) shares the same sequence, while a strong promoter (eno) has a truncated sequence (GGAG). Looking at the promoter sequences themselves does not provide insight into predictable markers of strength. The predicted ribosome binding sites, −35 regions, and −10 boxes vary amongst all of the promoters and do not exhibit discernible patterns amongst the weak or the strong promoters.

In comparison to *E. coli*, the promoters resulted in higher β-gal specific activity in *H. modesticaldum*. While *H. modesticaldum* and *E. coli* both have very similar ribosome binding sites, sharing AGGAGG, there must be more elements in the actual sequence of the promoter that dictate strength. Studies have shown that some clostridia are sensitive to the full sequence of the promoter, not just the −35 and −10 boxes, displaying drastic decreases in promoter strength when these sequences are altered [24]. Because *H. modesticaldum* is more closely related to *C. thermocellum*, the source of these promoters, than *E. coli* is, this may explain the higher activities in *H. modesticaldum* compared to *E. coli*. In addition, this sensitivity to the full promoter sequence may also explain why the activities of the promoters in *H. modesticaldum* are still different from those reported in *C. thermocellum* [16]. 

### 4.3. Inducible Promoters

We attempted to construct an inducible promoter for *H. modesticaldum* based on the anhydrotetracycline-inducible system developed and used for *C. acetobutylicum* [19]. To achieve this, we used several of the constitutive clostridial promoters to build various aspects of the system. The strongest promoter (P_gapDH_) was used to construct the inducible promoter, assuming that insertion of *tetO1* and any additional alterations would not strongly affect the promoter’s strength. The insertion of the *tetO* sequences into both -35/-10 boxes of gapDH only resulted in a 7% decrease in the correlated β-gal activity. 

It was unknown how active the promoter in front of the *tetR* gene needed to be to guarantee repression. Our initial study using a truncated version of the native *Clostridium perfringens* thiolase promoter (P_minipthl_) [4] to control the expression of *tetR* suggested that it was too strong for the system, as the P_gapDH/*tetO*_ promoter was strongly repressed but repression could not be relieved by aTc concentrations under which cells could grow (Figure S2). Therefore, we chose the next highest active promoter from our library of constitutive promoters (P_eno_). However, even when driving TetR expression from P_eno_, there was still residual P_gapDH/*tetO*_ promoter activity in the absence of aTc (at a similar level to one of the weakest constitutive promoters, e.g., 2638). While alleviation of the repression was strong, resulting in almost complete restoration of the activity of the promoter to that of the unmodified P_gapDH_, the breakthrough activity of this promoter renders it suboptimal for applications in which repression must be very tight (e.g., the expression of a potentially toxic enzyme). This system would be good, however, for high-level expression of proteins whose constitutive presence can be tolerated by *H. modesticaldum*.

Compared to the aTc system, the xylose-inducible promoter adapted from *C. perfringens* is simple and was easily introduced into *H. modesticaldum*. The *P_xyl_* promoter was inducible under increasing xylose concentrations from 0.03% to about 0.3%, and *H. modesticaldum* was shown to tolerate these concentrations of xylose without affecting their growth. The fact that this species does not metabolize xylose should ensure a constant inducer concentration. Thus, the *P_xyl_* promoter was inducible and highly tunable in the heliobacterial system. While the activity of the fully induced *P_xyl_* promoter is not the strongest, it should be useful for precise control of long-term expression of a gene of interest. The lack of detectable background activity in the absence of xylose makes this a promising system for the introduction of potentially toxic proteins, or for the complementation of a null mutation in an essential gene.

## Data Availability

Not applicable.

## Supplementary Materials

The following supporting information can be downloaded at: https://www.mdpi.com/article/10.3390/microorganisms10050876/s1, Table S1: sequences and annotations of promoters used in this study; Table S2: a list of primers used in this study; Table S3: a list of plasmids; Table S4: the plasmids constructed for this study with promoter activity determined in *H. modesticaldum* and *E. coli*; Figure S1: normalized β-gal activity in *H. modesticaldum*; and Figure S2: the activity of β-gal associated with different aTc plasmid constructs in *H. modesticaldum* with and without aTc inducer (200 µg L^−1^).
