# Supplementary material for: Examination of Genetic Control Elements in the Phototrophic Firmicute Heliomicrobium modesticaldum
Source: Microorganisms. 2022 Apr 22;10(5):876. doi: 10.3390/microorganisms10050876 (PMC9145376; doi:10.3390/microorganisms10050876)
Supplement: Supplementary file 1 [file microorganisms-10-00876-s001.zip › microorganisms-1681267-supplementary.pdf]

microorganisms

Supplementary materials

Table S1. The sequences and annotations of promoters used in this study.

Promoter	Sequence	Source
cbp_2	GAGTCGTGACTAAGAACGTCAAAGTAATTAACAATACAGCTATTTTTCTCATGCTTTT<br>ACCCCTTTCATAAAATTTAATTTTATCGTTATCATAAAAAATTATAGACGTTATATTG<br>CTTGCCGGGATATAGTGCTGGGCATTCGTTGGTGCAAAATGTTCGGAGTAAGGTGGA<br>TATTGATTTGCATGTTGATCTATTGCATTGAAATGATTAGTTATCCGTAAATATTAATT<br>AATCATATCATAAATTAATTATATCATAATTGTTTTGACGAATGAAGGTTTTTGGATA<br>AATTATCAAGTAAAGGAACGCTAAAAATTTTGGCGTAAAATATCAAAATGACCAC**T**<br>**TGAAT**TAATATGGTAAAGTAGA***TATAA***TATTTTGGTAAACATGCCTTCAGCAAGGTT<br>AGATTAGCTGTTTCCGTATAAATTAACCGTATGGTAAAACGGCAGTCAGAAAAATA<br>AGTCATAAGATTCCGTTATGAAAATATACTTCGGTAGTTAATAATAAGAGATATGAG<br>GTAAGAGATACAAGATAAGAGATATAAGGTACGAATGTATAAGATGGTGCTTTTAG<br>GCACACTAAATAAAAAACAAATAAACGAAAATTTTAAGGAGGACGAAAG	[1]
gapDH	TAATTACTGTATCTCTCTGGCA**TTGCCA**GGTTTTAATAAAGAT***TAAAAT***TATTGACTA<br>GAAATAAAAAAATTGTCCATAATATTAATGGACAAAAAAACAAAGAATTACATCA<br>AAGGAAGATAAAAATACTTTGTTAAAAAATTAATTATTTTTTATCTAAACTA**TTGAA**<br>**A**ATGAAAATAAAATAATA***TAAAAT***GAATCATAGTGCAAGAGATACTTGCCAGAGGA<br>TGAATATTTTACTGCATTCATGCTTTATGGCAGCTAATAGAGGCATTAAATTAAATTT<br>TAATTTACAATAGGAGGCGATATTAATG	[1]
gapDH/*tetO*	TAATTACTGTATCTCTCTGGCA**TTGCCA**actctatcattgatagag***TAAAAT***TATTGACTAGAA<br>ATAAAAAAATTGTCCATAATATTAATGGACAAAAAAACAAAGAATTACATCAAAG<br>GAAGATAAAAATACTTTGTTAAAAAATTAATTATTTTTTATCTAAACTA**TTGAAA**actc<br>tatcattgatagag***TAAAAT***GAATCATAGTGCAAGAGATACTTGCCAGAGGATGAATATTTT<br>ACTGCATTCATGCTTTATGGCAGCTAATAGAGGCATTAAATTAAATTTTAATTTACAA<br>TAGGAGGCGATATTAATG	[1],<br>This paper
eno	GGAAATATTAAAATGGAAATG**TTGAAA**AAATGTTTTAAGATGGGT***CATTAT***GGATA<br>AAATATACTATGGTTTTGCAATAAATGCTTTCTATTAATTGGACTTTGTGGTAATATG<br>GTAGAAGGATGCAGTGTTAATTTTTTAACATATAAAAATAAGCTATATGAAGGGAG<br>AATGGAGA	[1]
0815	ATAATATATATCCGTATTTTACAATATCCATACGAATATGGATTATTTTATTTTGTTAT<br>ATTTTTAACAAAAAAATTA**TTTAAA**CTTTTTCAATGGTGG***TATTAT***ATATATTGGTCA<br>CAGTTTCAATGAATC AAAAAATAAAGAGGTGTTTAAT	[1]
0966	TTCTTACTACTCCCTTTGCATCTACAGACAAATTTTTCTCTTTAACTTTTCTTGTAACC<br>GTATCCGCATACTTTTGTCATAAAGTAATTATAGATTATTATTACCGTCAAATCAAAT<br>AATAAATTTTATATCGCCAATTCT**TTGCAT**CCGCTTTAAAGTCTGTGC***TATAAT***TATA<br>TGGTTAAAATATCATAAAAAAAT AAAATAAAAGTTAAAAGGAACTGATTTT	[1]
2638	GATAAACAAAGGACGGTTCAGGGCTTCTGCTCATCCTACTCTGCATTGTAAAAAGGT<br>AGGATGAATTTTTATTTTTAATCTTATTGAAAAAAATTTTTGAAAATCGGTTTTA**TTA**<br>**AAA**AAAAGTGGGTATATT***TATAAT***AGTCAATTGATTGGTTAAAAAAATTTAAATAAG<br>CAAACAGAATAATAACAAAA GTAAGGAGGAATTTGTT	[1]
2926	AAAATATACAAAGGTTTCTTGTGTTTTTAATACCGTTATGT***TAATATAAT***GTAATA***TAT***<br>***ATT***T***TATAAT***AATATGTATGAGAGATAGTGTTTTGCTATATTGCTATAAAGAATGAGG<br>AGGGAACTAG	[1]
P_{xyl}*	TTTGATATTCCTCCTT***AAAATA***ATATTGTAATACTTTTT**ACACAA**AAATAAAAGGTT<br>ATTTTGCA**TTGACA**AAGATAATTAAATATTT***TATTAT***TAGTTCAtaagttagtttaatatactaa	[2]

	caaaaATAAAGCAAGTAAAATATACCTAAAATATAAAAAAATTAGGATAGGAAAA CGATAGTTATGAAGTGGCATTCAAGGAGGGAT GCAT

Predicted features are marked as follows: ribosome binding sites are underlined, -35 boxes are bolded, -10 boxes are italicized and bolded, and operators tetO1 (in gapDH 2tetO1) and xylO (in P_{xyl}) sequences are in lowercase.

*P_{xyl} contains two sets of -35/-10/RBS. The first RBS, -10, and -35 sequences are for the transcription of the *xylR* gene downstream of this set. The second RBS, -10, and -35 sequences are for the gene of interest, i.e., the gene whose transcription will be controlled by the promoter.

Table S2. List of primers used in this study. All primers were ordered from IDT.

Oligonucleotide	Sequence (5′ - 3′)	Purpose
adhB BglIII for	TAATCGAGATCTATGAAAGGTTTTGCAATGC	Amplification of *adhB**
adhB SalI rev	TAATCGGTCGACTTATGCTAATATTACAACAGGTTTG	
Bgal BglII for	TAATCGAGATCTATGAATGTGTTATCCTCAATTTG	Amplification of *lacZ**
Bgal SalI rev	TAATCGGTCGACCTAAACCTTCCCGGCTTC	
PgapDH PspOMI for	TAATACGGGCCCTAATTACTGTATCTCTCTGGC	Amplification of P_{gapDH}*
PgapDH BamHI rev	TAATACGGATCCTAATATCGCCTCCTATTGTAAATTAAA	
P2638 PspOMI for	TAATCGGGGCCCGATAAACAAAGGACGGTTC	Amplification of P_{2638}*
P2638 BamHI rev	TAATCGGGATCCAACAAATTCCTCCTTACTTTTG	
P2926 PspOMI for	TAATCGGGGCCCAAAATATACAAAGGTTTCTTGTG	Amplification of P_{2926}*
P2926 BamHI rev	ATAATCGGATCCAAAATATACAAAGGTTTCTTGTG	
Pcbp_2 PspOMI for	TAATCGGGGCCCGAGTCGTGACTAAGAACG	Amplification of P_{cbp_2}*
Pcbp_2 BamHI rev	TAATCGGGATCCCTTTCGTCCTCCTTAAAATTTTC	
Peno PspOMI for	TAATCGGGGCCCGGAAATATTAAAATGGAAATGTTG	Amplification of P_{eno}*
Peno BamHI rev	TAATCGGGATCCTCTCCATTCTCCCTTCATATAG	
P0966 PspOMI for	TAATCGGGGCCCTTCTTACTACTCCCTTTGC	Amplification of P_{0966}*
P0966 BamHI rev	TAATCGGGATCCAAAATCAGTTCCTTTTAACTTTTATT	
P0815 PspOMI for	TAATCGGGGCCCATAATATATATCCGTATTTTACAATATCC	Amplification of P_{0815}*
P0815 BamHI rev	TAATCGGGATCCATTAAACACCTCTTTATTTTTGATTC	
PgapDH BamHI for	AATCAGGATCCGGCCCTAATTACTGTATCTCTCTG	Amplification of gapDH-lacZ module to add to pUC19
PgapDH BglII for	AATCAAGATCTGGCCCTAATTACTGTATCTCTCTGGC	Amplification of $P_{gapDH/tetO}$-*lacZ* module to add to pMTL86251 downstream of P_{eno}-tetR
TetO1_1 for	CTATTGAAAACTCTATCATTGATAGAGTAAAATGAATC-ATAGTGC	Add *tetO1* to region between 2nd -35 and -10 in P_{gapDH}
TetO1_1 rev	ACTCTATCAATGATAGAGTTTTCAATAGTTTAGATAAAAAAT	
TetO1_2 for	TCCCTATCAGTGATAGATAAAATTATTGACTAGAAATAAAAAAATT GTCC	Add *tetO1* to region between 1st -35 and -10 in P_{gapDH}
TetO1_2 rev	TCAATAATTTTACTCTATCAATGATAGAGTTGGCAATGCCAGAGAG ATAC	
Eno rev EcoRI	ATTCGAATTCTCTCCATTCTCCCTTCATATAG	Amplification of P_{eno} to clone in front of *tetR*
Eno for BamHI	ATAATCGGATCCGGAAATATTAAAATGGAAATGTTGAA	
tetR for EcoRI	ATCTGAATTCATGTCTAGATTAGATAAAAGTAAAGTG	Amplification of *tetR* from *E. coli* to clone behind P_{eno}
tetR rev PspOMI	ATTTGGGCCCAACTCGACATCTTGGTTAC	

*Products were amplified from the appropriate pDGO plasmids [1] (**Table S3**).

Table S3. List of plasmids. All plasmids (with the exception of pDGO based plasmids) use pMTL86251 as the backbone.

Plasmid	Description	Source
pMTL86251	Modular shuttle vector for use in clostridial bacteria	[3]
pAL58	Plasmid containing adhB reporter gene driven by gapDH promoter	This paper
pAL61	Plasmid containing adhB reporter gene driven by 2926 promoter	This paper
pAL2B	Plasmid containing lacZ reporter gene driven by eno promoter	This paper
pAL60	Plasmid containing lacZ reporter gene driven by 2638 promoter	This paper
pAL62	Plasmid containing lacZ reporter gene driven by 2926 promoter	This paper
pAL64	Plasmid containing lacZ reporter gene driven by cbp_2 promoter	This paper
pAL66	Plasmid containing lacZ reporter gene driven by gapDH promoter	This paper
pAL731	Plasmid containing lacZ reporter gene driven by 0966 promoter	This paper
pAL751	Plasmid containing lacZ reporter gene driven by 0815 promoter	This paper
pAL88	Plasmid containing *lac*Z reporter gene driven by $P_{gapDH/\mathit{tetO}}$	This paper
pAL111	Plasmid containing *lac*Z driven by $P_{gapDH/\mathit{tetO}}$ and *tetR* driven by P_{eno}	This paper
pAL112	Plasmid containing *lac*Z driven by P_{gapDH} and *tetR* driven by P_{eno}	This paper
pALX3	Plasmid containing *lac*Z driven by P_{xyl} promoter and *xylR* repressor gene	This paper
pDGO89	Plasmid containing adhB reporter gene driven by 0815 promoter	Gift from Dan Olson [1]
pDGO95	Plasmid containing *lac*Z reporter gene driven by gapDH promoter	Gift from Dan Olson [1]
pDGO102	Plasmid containing *lac*Z reporter gene driven by eno promoter	Gift from Dan Olson [1]
pDGO105	Plasmid containing *lac*Z reporter gene driven by 0966 promoter	Gift from Dan Olson [1]
pDGO106	Plasmid containing *lac*Z reporter gene driven by 2638 promoter	Gift from Dan Olson [1]
pDGO108	Plasmid containing *lac*Z reporter gene driven by 2926 promoter	Gift from Dan Olson [1]
pDGO117	Plasmid containing *lac*Z reporter gene driven by cbp_2 promoter	Gift from Dan Olson [1]

Table S4. Plasmids constructed for this study with promoter activity determined in *H. modesticaldum* and *E. coli*.

Plasmid	Promoter	Reporter gene	Detected activity in *H. modesticaldum* (U/mg protein)	Detected activity in *E. coli* (U/mg protein)
pAL2B	eno	*lacZ*	0.60 ± 0.019	0.17 ± 0.0050
pAL60	2638	*lacZ*	0.067 ± 0.0042	0.037 ± 0.0028
pAL62	2926	*lacZ*	0.39 ± 0.011	0.39 ± 0.011
pAL64	cbp_2	*lacZ*	0.64 ± 0.014	0.39 ± 0.011
pAL66	gapDH	*lacZ*	0.79 ± 0.027	0.25 ± 0.0069
pAL731	0966	*lacZ*	0.11 ± 0.0044	0.11 ± 0.0045
pAL751	0815	*lacZ*	0.10 ± 0.0044	0.013 ± 0.00077

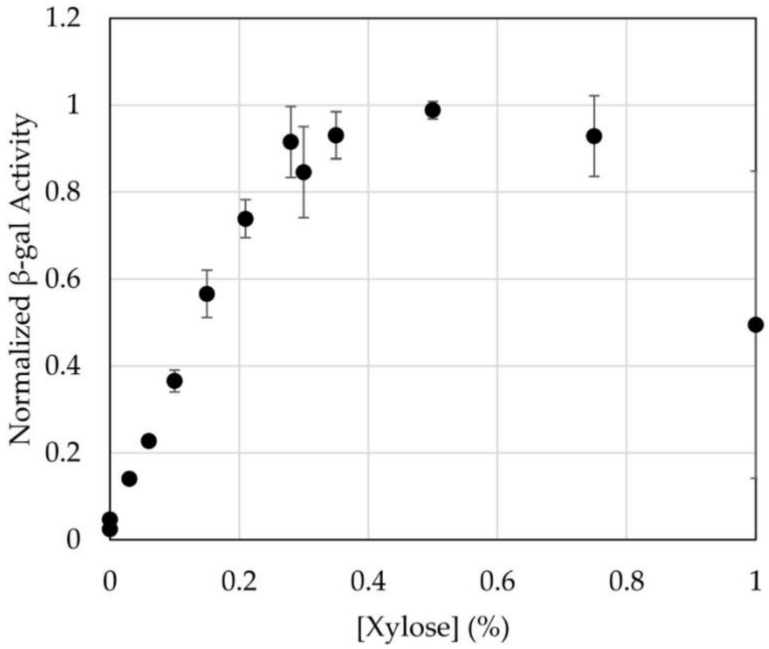

Figure S1. The normalized β-gal activity in *H. modesticaldum*. The activity of β-gal in *H. modesticaldum* transformed with pALX3 and grown with xylose at the indicated concentrations, normalized to the maximal activity obtained. The activity assays were performed on two separate days; therefore, data sets for each biological triplicate were scaled according to the activity at 0.35% and then normalized to the maximum activity. Points represent the average of normalizations, and error bars represent standard deviation.

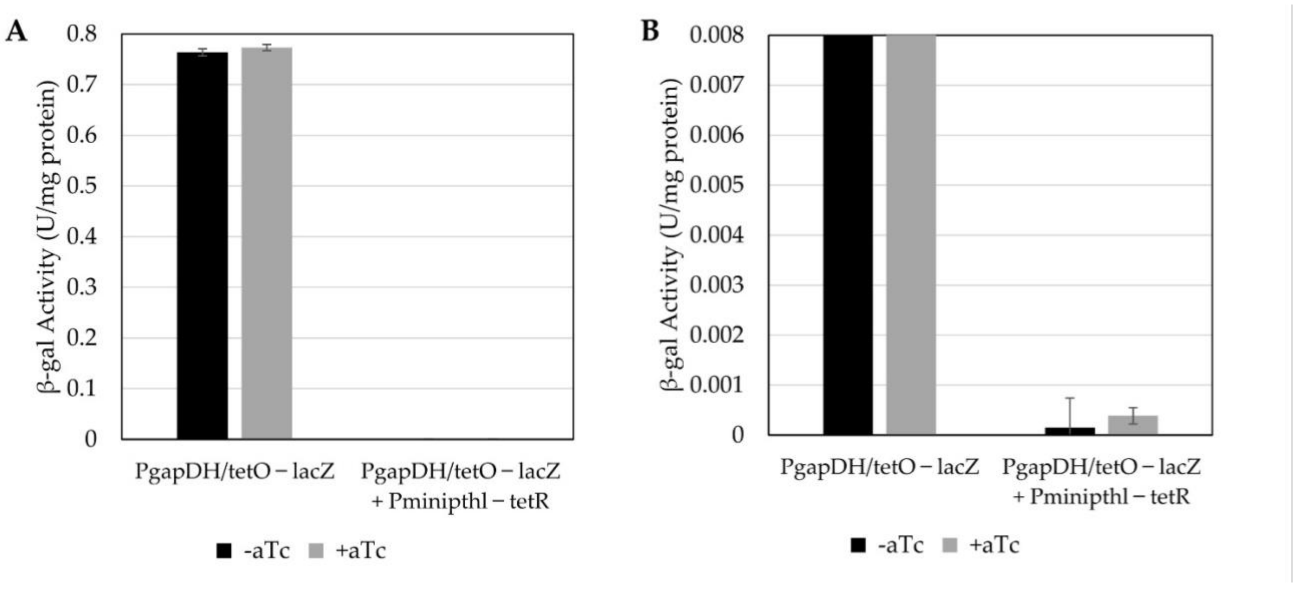

Figure S2. The activity of β-gal associated with different aTc plasmid constructs in *H. modesticaldum* with and without aTc inducer (200 µg L^{-1}). The constructs include $P_{gapDH/\mathit{tetO}}$ controlling *lacZ* (left), and a second construct including the *tetR* repressor controlled by $P_{minipthl}$ (right). Data represent the average rate of biological and technical triplicate; error bars represent the average standard deviation of each set of biological replicates. The ordinate scale is expanded 100-fold in plot **B** in order to see the activity of cells harboring the second construct.

References

1. Olson, D.G.; Maloney, M.; Lanahan, A.A.; Hon, S.; Hauser, L.J.; Lynd, L.R. Identifying Promoters for Gene Expression in *Clostridium Thermocellum*. *Metab. Eng. Commun.* **2015**, *2*, 23–29, doi:10.1016/j.meteno.2015.03.002.
2. Nariya, H.; Miyata, S.; Kuwahara, T.; Okabe, A. Development and Characterization of a Xylose-Inducible Gene Expression System for *Clostridium Perfringens* †.**2011**, *77*, 8439–8441, doi:10.1128/AEM.05668-11.
3. Heap, J.T.; Pennington, O.J.; Cartman, S.T.; Minton, N.P. A Modular System for Clostridium Shuttle Plasmids. *J. Microbiol. Methods* **2009**, *78*, 79–85, doi:10.1016/j.mimet.2009.05.004.
